# Prevalence and factors associated with concomitant bacteremia among adults admitted with severe malaria at Kayunga Regional Referral Hospital, Uganda

**DOI:** 10.1186/s12936-026-05793-4

**Published:** 2026-01-20

**Authors:** Farah Dubad Abdi, Abishir Mohamud Hirsi, Mutaz Ali, Abdifatah Hersi Karshe, Abdisalam Ahmed Sandeyl, Abdisamed Guled Hersi, Abdirizak Abdinasir Yusuf, Hailemariam Kassahun Bekele, Abdifitah Abdullahi Mohamed, Mohamed Jayte, Agwu Ezera

**Affiliations:** 1 Department of Internal Medicine, Faculty of Clinical Medicine and Dentistry, Kampala International University, Ishaka, Bushenyi, Ishaka, Uganda; 2 Department of Microbiology and Immunology, Faculty of Biomedical Sciences, Kampala International University, Ishaka, Bushenyi, Ishaka, Uganda; 3 Department of Microbiology and Parasitology, School of Medicine and Pharmacy College of Medicine and Health Sciences University of Rwanda, Kigali, Rwanda

**Keywords:** Prevalence, Bacteremia, Severe malaria, Bacterial profile, Risk factors, Hyperparasitaemia, Susceptibility patterns, Uganda

## Abstract

**Background:**

Malaria-bacteremia co-infection significantly increases mortality and the risk of ICU admission. Diagnostic overlap with bacterial infections often results in misdiagnosis, impacting outcomes. While pediatric data exists, adult studies in Uganda are limited. This study aimed to determine the prevalence, bacterial isolates, and associated factors of bacteremia in adults with severe malaria at Kayunga Regional Referral Hospital.

**Methods:**

A cross-sectional study enrolled 207 adults with severe malaria. Blood samples were cultured, and isolates tested for antimicrobial susceptibility. Sociodemographic, clinical, and laboratory data were collected using structured tools. Logistic regression in SPSS version 26 was done to determine the significant factors. The outcome predicted was the presence of bacterium. P < 0.05 was considered significant.

**Results:**

Of the 207 participants, 14.5% had bacteremia. Central nervous system (CNS) symptoms, low peripheral oxygen saturation (SPO2), hyperparasitaemia, and leucocytosis were significantly associated with bacteremia. *Salmonella typhi* (33.3%), *Staph aureus* (30%), and *Streptococcus* spp. (16.7%) were the most common isolates. Ciprofloxacin and penicillin derivatives showed strong coverage.

**Conclusion:**

The prevalence of bacteremia among patients with malaria was high, seen in over one of every seven patients with malaria. Malaria patients with CNS symptoms, low peripheral oxygen saturation, malaria hyperparasitaemia and leucocytosis should be considered to be at high risk for bacteremia. If bacteria co-infection is suspected among patients with malaria, in the absence of culture and sensitivity results, a combination of ciprofloxacin and a penicillin can be considered since these two can provide an acceptable cover of the most common isolates, yet readily available in our resource limited setting.

## Introduction

The 2024 World Malaria Report [1] highlights increasing global cases of malaria (263 million in 2023) but declining deaths (597,000 in 2023), with the WHO African Region bearing the highest burden (95% of cases/deaths), especially children under 5, while facing challenges like insecticide resistance [1]. Uganda continues to bear a high malaria burden, particularly in regions like Kayunga, where intermittent rains create ideal breeding grounds for malaria vectors [2, 3].

Severe malaria remains a major cause of hospitalization in Africa according to the 2024 World Malaria Report. While most studies in East Africa have focused on children, adult data are limited. For instance, studies in Kenya and Tanzania reported bacteraemia prevalence rates of 11.7% and 9.3%, respectively, among children with severe malaria [4, 5].

Co-infection with malaria and bacterial pathogens is associated with poor outcomes, including increased mortality and prolonged hospitalization. However, overlapping clinical features between malaria and bacterial infections, coupled with limited diagnostic capacity, often lead to underdiagnosis and mismanagement [6].

In Uganda, poor hygiene and sanitation may further elevate the risk of bacteremia in malaria patients [7]. Despite this, limited research exists on bacteremia among adults with severe malaria. This study therefore aimed to assess the prevalence, bacterial profile, and associated factors of bacteremia in adults with severe malaria at Kayunga Regional Referral Hospital.

## Methods

### Study design

This cross-sectional study was conducted among adults diagnosed with severe malaria at Kayunga Regional Referral Hospital (KRRH). Blood samples were collected for bacteremia testing, and bacterial isolates were identified along with their antimicrobial susceptibility pattern.

### Study setting

Kayunga Regional Referral Hospital, located in Kayunga District, serves as a referral hospital for neighboring areas. It has a 254-bed capacity and multiple departments, including internal medicine. The hospital also functions as a teaching site for Kampala International University and is equipped to perform culture and sensitivity tests.

### Study population

The study population consisted of adults aged ≥ 18 years who were admitted to the emergency department or inpatient medical wards with severe malaria. Screening for malaria was based on the presence of compatible clinical features, including fever or history of fever, chills, headache, jaundice, altered mental status, prostration, or signs of organ dysfunction. Malaria diagnosis was confirmed using both a histidine-rich protein 2 (HRP-2) rapid diagnostic test (Standard Diagnostics Bioline Malaria Ag P.f) and Giemsa-stained peripheral blood smear microscopy for parasite detection and quantification. Severe malaria was defined as malaria with any severity feature including presence of one or more features such as impaired consciousness, prostration, respiratory distress, shock, acute kidney injury, jaundice, severe anemia (Hb < 7 g/dL), hyperparasitemia (> 250,000 parasites/μL), or evidence of bleeding or disseminated intravascular coagulation in line with the Who criteria.

### Eligibility criteria

Adults with confirmed severe malaria who consented to participate were included for participation, while patients who had taken antibiotics within the two weeks prior to presentation were excluded.

### Sample size

Using OpenEpi and findings from Chau et al. [8] a sample size of 188 was calculated based on a reported risk ratio of 8.1 for bacteremia in patients with > 20% parasitemia. With an additional 10% to account for non-response, the final sample size was 207.

### Sampling technique

Participants were enrolled consecutively until the target sample size was achieved.

### Data collection tools

Data were collected using a structured questionnaire in both English and the local language. Sociodemographic and clinical data were extracted from medical records. Diagnostic tools included a rapid HIV test, HIV testing was performed using the Uganda National HIV Testing Algorithm, consisting of Determine™ HIV-1/2 as the screening test and STAT-PAK^®^ for confirmation. Uni-Gold™ HIV was used as the tie-breaker where results were discordant. Littman Class III stethoscope was used for auscultation, digital thermometer used to take temperature, and Sysmex XN-1000 hematology analyzer used for blood count. Blood for culture was drawn using sterile technique and processed in both aerobic (BD Peds Plus/F) and anaerobic (VersaTREK Redox) bottles.

### Measurement of study variables

#### Dependent variables

*Bacteraemia* Presence of viable bacteria in blood, confirmed by positive blood culture.

Physiological and laboratory variables were classified using standard cut-off values. Body mass index (BMI) was categorized using WHO criteria [9]: underweight (< 18.5 kg/m^2^), normal (18.5–24.9 kg/m^2^), overweight (25.0–29.9 kg/m^2^), and obese (≥ 30 kg/m^2^) [9] Blood pressure was classified following American Heart Association 2017 guidelines [10]. Respiratory rate was categorized as tachypnea if > 20 breaths/min, and pulse rate as tachycardia if > 100 beats/min. Peripheral oxygen saturation (SpO₂) was considered low if < 94%. Glasgow Coma Scale (GCS) was considered reduced if < 15. Laboratory variables were classified using site-standard reference ranges.

For blood culture, 8–10 mL of venous blood was aseptically collected before antibiotic administration and inoculated into a single aerobic BACT/ALERT^®^ FA Plus bottle. The bottles were incubated in an automated BACT/ALERT^®^ VIRTUO Culture System for up to 5 days. The instrument automatically flagged positive bottles, which were then subjected to Gram staining, subculture on blood agar, chocolate agar, and MacConkey agar, and incubated at 35–37 °C. Organism identification was performed using standard biochemical tests and API^®^ identification systems. Suspected *Salmonella* isolates underwent serogrouping and serotyping using commercial antisera to differentiate *Salmonella Typhi* from non-typhoidal serovars.

Antimicrobial susceptibility testing (AST) was performed by the Kirby-Bauer disk diffusion method according to the Clinical and Laboratory Standards Institute (CLSI) M100, 2023 guidelines. Cefoxitin disc testing was used for Methicillin-resistant Staphylococcus aureus (MRSA) detection. Where azithromycin or ceftriaxone resistance was detected in *Salmonella Typhi*, minimum inhibitory concentration (MIC) confirmation was performed using E-test strips (bioMérieux).

Internal quality control included *E. coli* ATCC 25922, *S. aureus* ATCC 25923, and *Salmonella Typhi* ATCC 9992. The laboratory participates in an ongoing external quality assessment scheme supervised by the Uganda National Health Laboratory Services.

Culture significance was determined based on clinical context, organism type, and growth characteristics. Contaminants were defined as typical skin flora (e.g., coagulase-negative staphylococci, Corynebacterium spp., or Bacillus spp.) isolated in a single bottle without clinical evidence of infection and were excluded from analysis.

*Bacterial profile* Refers to the identified organisms and their antibiotic susceptibility.

### Independent variables

*Age* The length of time a person has lived, typically measured in years from birth.

*Sex* A biological classification (male, female, or intersex) based on physical characteristics such as chromosomes and reproductive anatomy**.**

*Level of Education* The highest degree or formal schooling a person has completed (e.g., high school, bachelor’s degree, PhD).

*Occupation* A person’s job, profession, or primary work role (e.g., teacher, engineer, nurse).

All are key demographic variables used in research, surveys, and policy-making.

*Convulsions*: Sudden, uncontrolled muscle contractions (seizures) often with jerking movements and loss of consciousness.

*Respiratory Distress*: Difficulty breathing, characterized by rapid breathing, gasping, or labored breaths.

*Jaundice*: Yellowing of the skin and eyes due to high bilirubin levels (liver dysfunction or excessive red blood cell breakdown).

*CNS Symptoms* Symptoms affecting the central nervous system (brain/spinal cord), such as confusion, headaches, seizures, or paralysis.

*Hepatomegaly* Abnormal enlargement of the liver.

*Splenomegaly* Abnormal enlargement of the spleen.

### Data quality control

A pretest of the questionnaire was conducted to ensure clarity. Interviews were conducted in the local language to reduce recall bias. All instruments used were regularly calibrated. The principal investigator reviewed completed questionnaires daily and provided training and supervision to data collectors. A physician oversaw clinical aspects.

### Data management and analysis

Completed forms were verified at the point of collection. No post-collection changes were made. Data were securely stored in password-protected files. Final datasets were cleaned, coded, and analyzed using IBM SPSS version 26.0 (Armonk, NY: IBM Corp). The prevalence of bacteremia was determined by dividing the number of participants who had the growth by the total number of participants. A frequency and percentage were used to express it. Binary logistic regression was used to investigate the factors linked to bacteremia. We reported both the unadjusted (crude) odds ratios along with their respective confidence intervals (CI) and the adjusted odds ratios. In the multivariable model, a variable was considered significant if P is less than 0.05. The proportion of the most common bacterial isolates that were sensitive to specific antibiotics were calculated as fractions of sensitive organisms for a specific antibiotic over the total number of participants with the bacteremia.

## Results

### Characteristics of the study participants

A total of 207 adults with severe malaria were enrolled. Over half (51.7%) were aged 18–45 years, and males slightly outnumbered females (51.2% vs. 48.8%). Most participants (69.1%) had symptoms lasting more than three days before presentation. Hyperparasitaemia was observed in 38.2%, and 25.6% had leucocytosis. Comorbidities (HIV, diabetes mellitus, sickle cell disease and tuberculosis) were classified as previously diagnosed and on treatment based on documented medical records; participants without known history underwent confirmatory testing where applicable (HIV rapid test; fasting glucose for diabetes; review of laboratory and radiological records for tuberculosis and sickle cell disease). Detailed characteristics are presented in Table 1.

**Table 1:** Characteristics of study participants

Characteristic	Frequency	Percentage
Age (years)		
18–45	107	51.7
46–65	44	21.3
66 +	56	27.0
Sex		
Male	106	51.2
Female	101	48.8
Residence		
Urban	58	28.0
Rural	149	72.0
Marital status		
Married	78	37.7
Single	117	56.5
Widow	12	5.8
Religion		
Christian	177	85.5
Muslim	30	14.5
Education level		
primary	73	35.3
Secondary	110	53.1
Tertiary	24	11.6
Occupation		
Formal employment	8	3.9
Peasant	63	30.4
Business	53	25.6
Student	75	36.2
Other	8	3.9
Smoking		
No	202	97.6
Yes	5	2.4
Alcohol use		
No	172	83.1
Yes	35	16.9
Chronic illness		
No	148	71.5
Yes	59	28.5
HIV		
No	183	88.4
Yes	24	11.6
Diabates		
No	187	90.3
Yes	20	9.7
Sickle cell disease		
No	199	96.1
Yes	8	3.9
Tuberculosis		
No	198	95.7
Yes	9	4.3
Duration of symptoms		
≤ 3	64	30.9
4 +	143	69.1
CNS symptoms		
No	112	54.1
Yes	95	45.9
Respiratory symptoms		
No	64	30.9
Yes	143	69.1
Jaundice		
No	125	60.4
Yes	82	39.6
BMI category		
Normal	124	59.9
underweight	9	4.3
Overweight	67	32.4
Obese	7	3.4
Blood pressure		
Normal	150	72.5
Low	12	5.8
Elevated	14	6.8
High	31	15.0
Respiratory rate		
Normal	27	13.0
Tachypnea	180	87.0
Pulse rate		
Normal	97	46.9
Tachycardia	110	53.1
SPO2		
Low	52	25.1
Normal	155	74.9
GCS		
< 15	114	55.1
Normal	93	44.9
Hepatomegaly		
No	181	87.4
Yes	26	12.6
Splenomegaly		
No	171	82.6
Yes	36	17.4
Hyperparasitaemia		
No	128	61.8
Yes	79	38.2
Hemoglobin		
Severe anemia	10	4.8
Moderate anemia	93	44.9
Mild anemia	39	18.8
Normal	65	31.4
WBC		
Normal	154	74.4
Leucocytosis	53	25.6
Platelets		
Thrombocytopenia	34	16.4
Normal	173	83.6

### Prevalence of bacteraemia among adults presenting with severe malaria

**Fig. 1:**
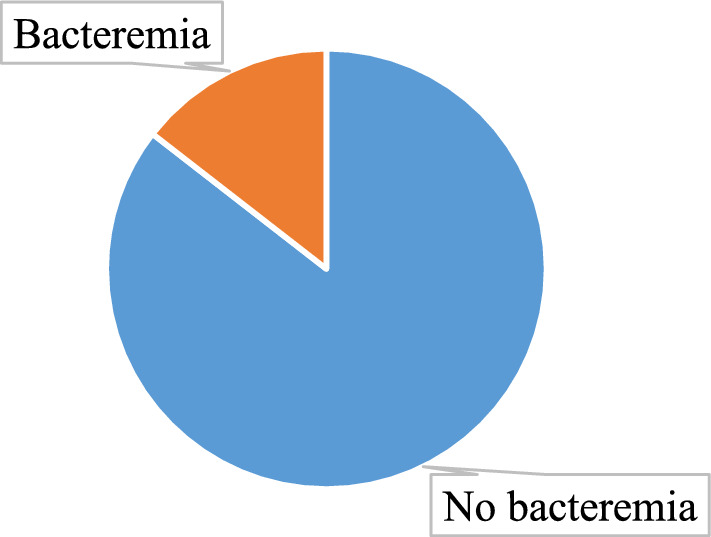
Prevalence of bacteraemia among adults presenting with severe malaria

Among the 207 patients with severe malaria enrolled into the study, only 30 had bacterial growth, showing an incidence of 14.5% with a corresponding 95% confidence interval of 9.7–19.3% as shown in Fig. 1 below.

### Factors associated with bacteraemia among adults presenting with severe malaria

Variables with p-values < 0.2 from bivariate analysis were considered for multivariable analysis. These included age category, sex, diabetes mellitus, sickle cell disease, tuberculosis, duration of illness, central nervous system (CNS) symptoms, SPO2, Glasgow Coma Scale (GCS), hyperparasitaemia, and leucocyte count. Full bivariate results are presented in Table 2 below.

**Table 2:** Bivariable analysis of factors associated with bacteraemia among adults presenting with severe malaria

Characteristic	No bacteremia N = 177	Bacteremia N = 30	Bivariable analysis		
			cOR	95% CI	P value
Age (years)					
18–45	98 (55.4)	9 (30.0)	Ref		
46–65	38 (21.5)	6 (20.0)	1.719	0.573–5.159	0.334
66 +	41 (23.2)	15 (50.0)	3.984	1.614–9.830	**0.003**
Sex					
Male	87 (49.2)	19 (63.3)	1.787	0.804–3.972	**0.154**
Female	90 (50.8)	11 (36.7)	Ref		
Residence					
Urban	52 (29.4)	6 (20.0)	Ref		
Rural	125 (70.6)	24 (80.0)	1.664	0.643–4.308	0.294
Education level					
Primary	61 (34.5)	12 (40.0)	1.377	0.354–5.359	0.644
Secondary	95 (53.7)	15 (50.0)	1.105	0.293–4.165	0.882
Tertiary	21 (11.9)	3 (10.0)	Ref		
Smoking					
No	173 (97.7)	29 (96.7)	Ref		
Yes	4 (2.3)	1 (3.3)	1.491	0.161–13.819	0.725
Alcohol use					
No	146 (82.5)	26(86.7)	Ref		
Yes	31 (17.5)	4(13.3)	0.725	0.236–2.225	0.573
Chronic illness					
No	125(70.6)	23(76.7)	Ref		
Yes	52(29.4)	7(23.3)	0.732	0.296–1.810	0.499
HIV					
No	157 (88.7)	26 (86.7)	Ref		
Yes	20 (11.3)	4 (13.3)	1.208	0.382–3.818	0.748
Diabates					
No	162 (91.5)	25(83.3)	Ref		
Yes	15 (8.5)	5(16.7)	2.160	0.722–6.465	**0.169**
Sickle cell disease					
No	172 (97.2)	27(90.0)	Ref		
Yes	5(2.8)	3(10.0)	3.822	0.863–16.921	**0.077**
Tuberculosis					
No	171 (96.6)	27 (90.0)	Ref		
Yes	6 (3.4)	3 (10.0)	3.167	0.747–13.421	**0.118**
Duration of symptoms					
≤ 3	60 (33.9)	4(13.3)	Ref		
4 +	117 (66.1)	26(86.7)	3.333	1.112–9.991	**0.032**
CNS symptoms					
No	110 (62.1)	2 (6.7)	Ref		
Yes	67 (37.9)	28 (93.3)	2.985	1.304–9.603	** < 0.001**
Respiratory symptoms					
No	55 (31.1)	9 (30.0)	Ref		
Yes	122 (68.9)	21 (70.0)	1.052	0.453–2.445	0.906
Jaundice					
No	105 (59.3)	20 (66.7)	Ref		
Yes	72 (40.7)	10 (33.3)	0.729	0.322–1.649	0.448
BMI category					
Normal	110 (62.1)	14 (46.7)	Ref		
underweight	7 (4.0)	2 (6.7)	2.245	0.424–11.889	0.342
Overweight	54 (30.5)	13 (43.3)	1.892	0.831–4.304	0.229
Obese	6 (3.4)	1 (3.3)	1.310	0.147–11.687	0.809
Blood pressure					
Normal	132 (74.6)	18 (60.0)	Ref		
Low	10 (5.6)	2 (6.7)	1.467	0.297–7.236	0.638
Elevated	10 (5.6)	4 (13.3)	2.933	0.832–10.339	0.294
High	25 (14.1)	6 (20.0)	1.760	0.636–4.871	0.276
Respiratory rate					
Normal	22 (12.4)	5 (16.7)	Ref		
Tachypnea	155 (87.6)	25(83.3)	0.710	0.246–2.046	0.526
Pulse rate					
Normal	83 (46.9)	14 (46.7)	Ref		
Tachycardia	94 (53.1)	16 (53.3)	1.009	0.465–2.192	0.982
SPO2					
Low	38 (21.5)	14 (46.7)	3.201	1.435–7.137	**0.004**
Normal	139 (78.5)	16 (53.3)	Ref		
GCS					
< 15	89 (50.3)	25 (83.3)	4.944	1.811–13.498	**0.002**
Normal	88 (49.7)	5 (16.7)	Ref		
Hepatomegaly					
No	153 (86.4)	28 (93.3)	Ref		
Yes	24 (13.6)	2 (6.7)	0.455	0.102–2.036	0.303
Splenomegaly					
No	148 (83.6)	23 (76.7)	Ref		
Yes	29 (16.4)	7 (23.3)	1.553	0.610–3.956	0.356
Hyperparasitaemia					
No	118 (66.7)	10 (33.3)	Ref		
Yes	59 (33.3)	20 (66.7)	4.000	1.760–9.090	**0.001**
Hemoglobin					
Severe anemia	8 (4.5)	2 (6.7)	1.375	0.254–7.449	0.712
Moderate anemia	82 (46.3)	11 (36.7)	0.738	0.293–1.855	0.518
Mild anemia	32 (18.1)	7 (23.3)	1.203	0.417–3.471	0.732
Normal	55 (31.1)	10 (33.3)	Ref		
WBC					
Normal	138 (78.0)	16(53.3)	Ref		
Leucocytosis	39 (22.0)	14(46.7)	3.096	1.390–6.894	**0.006**
Platelets					
Thrombocytopenia	29 (16.4)	5 (16.7)	Ref		
Normal	148 (83.6)	25 (83.3)	0.980	0.346–2.770	0.969

*cOR* Crude odds ratio, *CI* Confidence interval, *HIV*  Human immunodeficiency virus, *BMI* Body mass index, *CNS* central nervous system, *SPO2* peripheral oxygen saturation, *GCS* Glasgow coma scale, *WBC* white blood count.

During multivariable analysis, the predictor determinants related to bacteremia were presence of CNS symptoms (aOR = 2.5449, CI = 1.456–6.998, P < 0.001), having low peripheral oxygen saturation (aOR = 4.389, CI = 1.591–12.106, P = 0.004), presence of malaria hyperparasitaemia (aOR = 3.816, CI = 1.166–12.494, P = 0.027) and presence of leucocytosis (aOR = 2.472, CI = 1.963–6.342, P = 0.046).

### Bacteria isolates associated with bacteraemia among adults presenting with severe malaria

**Fig. 2:**
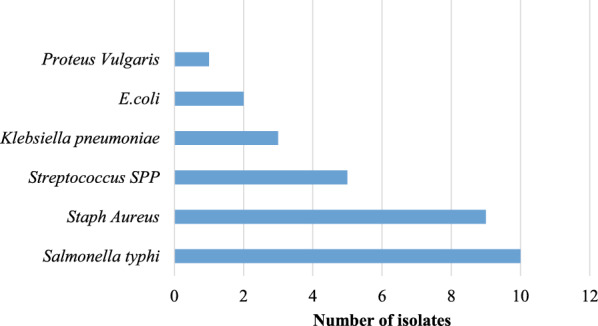
Bacteria isolates associated with bacteraemia among adults presenting with severe malaria

Among the 30 participants in whom bacteremia growth was observed, the most common organism isolated was *Salmonella typhi*, accounting for 33.3% of the isolates, followed by *Staph aureus* accounting for 30.0% and *Streptococcus SPP* in 16.7% (Fig. 2). Among the five streptococcal isolates recovered, two were identified as *Streptococcus pneumoniae* based on optochin susceptibility and bile solubility testing, one was *Streptococcus pyogenes* (Group A), and one was *Streptococcus agalactiae* (Group B) (Table 3). The remaining isolate was classified as viridans group streptococcus and considered a contaminant since it was isolated from a single culture bottle and lacked corresponding clinical features of true infection (Table 4).

**Table 3:** Multivariable analysis of factors associated with bacteraemia

Characteristic	Multivariable aOR	95% CI	P-value
Age (years)			
18–45 (Ref)	Ref		
46–65	1.136	0.203–2.666	0.640
66 +	1.788	0.517–6.178	0.359
Sex			
Male	1.504	0.552–4.096	0.425
Female (Ref)	Ref		
Diabetes			
No (Ref)	Ref		
Yes	1.597	0.109–3.276	0.553
Sickle cell disease			
No (Ref)	Ref		
Yes	1.401	0.504–8.591	0.144
Tuberculosis			
No (Ref)	Ref		
Yes	1.091	0.118–8.336	0.593
Duration of symptoms			
≤ 3 days (Ref)	Ref		
> 3 days	2.334	0.640–8.506	0.199
CNS Symptoms			
No (Ref)	Ref		
Yes	2.5449	1.456–6.998	< 0.001
SPO2			
Normal (Ref)	Ref		
Low	4.389	1.591–12.106	0.004
GCS			
Normal (Ref)	Ref		
< 15	1.255	0.349–4.510	0.072
Hyperparasitaemia			
No (Ref)	Ref		
Yes	3.816	1.166–12.494	0.027
Leucocytosis			
No (Ref)	Ref		
Yes	2.472	1.963–6.342	0.046

*cOR* Crude Odds Ratio, *aOR* Adjusted Odds Ratio, *CI* Confidence Interval, *CNS* Central Nervous System, *SPO2* Peripheral Oxygen Saturation, *GCS* Glasgow Coma Scale, *WBC* White Blood Cell Count.

**Table 4:** Susceptibility patterns of bacterial isolates in severe malaria patients with bacteremia

Antibiotic	*Salmonella typhi* (N = 10)	*Staph aureus (N* = *9)*	*Streptococcus spp (N* = *5)*	*Klebsiella pneumoniae (N* = *3)*	*E. coli (N* = *2)*	*Proteus vulgaris (N* = *1)*
Ciprofloxacin	S 90.0% I 10.0%	S 55.6% I 22.2% R 22.2%		S 66.7% I 33.3%		S 100.0%
Levofloxacin	S 90.0% I 10.0%		S 80.0% I 20.0%			
Doxycycline		S 77.8% I 22.2%				
Penicillin			S 80.0% I 20.0%			
Imipenem				S 100.0%	S 100.0%	S 100.0%
Chloramphenicol	S 70.0% I 30.0%				S: 100.0%	S: 100.0%
Trimethoprim-sulfamethoxazole				I 33.3% R 66.7%		R 100.0%
Gentamycin		R 100.0%		I 33.3% R 66.7%	I 50.0% R 50.0%	R 100.0%
Ceftriaxone	S 70.0% R 30.0%			S 100.0%	S 50.0% I 50.0%	I 100.0%
Amikacin		I 77.8% R 22.2%		I 33.3% R 66.7%	I 50.0% R 50.0%	
Vancomycin			I 100.0%			
Cefepime			S 100.0%			
Azithromycin	S 60.0% R 40.0%	S 77.8% I 22.2%				
Clindamycin		S 77.8% I 22.2%	S 100.0%			
Cefoxitin		S 88.9% I 11.1%			S 50.0% I 50.0%	
Tetracycline			I 20.0% R 80.0%			
Minocycline		R 100.0%				
Oxacillin		I 77.8% R 22.2%				
Cefalexin					S 50.0% I 50.0%	
Ampicillin				I 66.7% R 33.3%	I 50.0% R 50.0%	
Ceftazidime-Tazobactam				S 66.7% I 33.3%	I 50.0% R 50.0%	
Erythromycin			S 80.0% I 20.0%			

S (Susceptible): The bacteria are sensitive to the antibiotic and can be treated effectively.

I (Intermediate): The antibiotic may be effective at higher doses or in specific body sites.

R (Resistant): The bacteria are not inhibited or killed by the antibiotic at normal doses.

## Discussion

This study aimed to determine the prevalence, bacterial profile, and factors associated with concomitant bacteremia among adults admitted with severe malaria at Kayunga Regional Referral Hospital. Our findings provide insights relevant to clinical management and align with, or differ from, previously published literature.

### Prevalence of bacteremia

Among the 207 patients enrolled, bacteremia was identified in 30 individuals, yielding a prevalence of 14.5%. This is relatively high compared to the pooled prevalence of 7.6% reported in a meta-analysis by Wilairatana et al. [11], and far higher than 0.3% reported in a retrospective study among malaria patients in Sweden [11]. This discrepancy may be attributed to differences in socioeconomic factors, healthcare access, and nutritional status, as noted by Ricci [12], who emphasized the role of poverty and undernutrition in infectious disease susceptibility.

Our findings are comparable to those by Hanson et al. [13] and Nyein et al. [14], who reported bacteremia prevalence rates of 10% and 13%, respectively, among hospitalized malaria patients. The similarity may be due to comparable clinical settings and study populations. Conversely, Ukaga et al. [15] reported a much higher prevalence (35.2%), possibly due to historical differences in economic conditions and the predominance of Salmonella infections linked to poor hygiene.

### Factors associated with bacteremia

Multivariable analysis identified four independent predictors of bacteremia: presence of CNS symptoms, low peripheral oxygen saturation, malaria hyperparasitemia, and leukocytosis.

CNS symptoms increased the odds of bacteremia by over 2.5 times. Donnelly et al. [16] explain that falciparum-infected erythrocytes adhere to vascular endothelium, causing microvascular occlusion, hypoxia, and GI epithelial damage that permits bacterial translocation. Our study found that 93.3% of bacteremic patients had CNS symptoms, aligning with this mechanism. Additionally, White [17] associated falciparum malaria with seizures even in uncomplicated cases.

Low oxygen saturation increased the odds of bacteremia by over four times. White [18] and Adebola et al. [19] discuss how malaria-induced anemia and respiratory complications such as aspiration and airway obstruction contribute to hypoxia. In our study, 70% of bacteremic participants had respiratory issues, with 83.3% exhibiting tachypnea.

Hyperparasitemia was significantly associated with bacteremia (OR ≈ 4). According to Takem et al. [20], hemolysis in hyperparasitemia elevates iron levels and impairs neutrophil function, promoting bacterial proliferation. Chau et al. [8] found higher rates of bacteremia in patients with parasitemia > 20%.

Leukocytosis was also associated with a twofold increase in the odds of bacteremia. Babatunde and Adenuga [21] suggest that while neutrophils combat malaria via phagocytosis and ROS production, malaria parasites can suppress antimicrobial responses, heightening susceptibility to secondary infections such as non-typhoidal Salmonella.

### Bacterial profile

*Salmonella typhi* was the most frequently isolated organism (33.3%), followed by *Staphylococcus aureus* (30.0%) and *Streptococcus* spp. (16.7%). This is consistent with the findings of Wilairatana et al. [11], who reported similar predominant isolates in a meta-analysis of bacteremia in malaria patients.

Other studies also support our findings. For instance, Piyaphanee et al. [22] reported two cases of Salmonella bacteremia in P. vivax-infected patients in Thailand. Bhattacharya et al. [23] found that Gram-negative organisms, including *S. Typhi*, were prevalent among febrile patients in Kolkata. Similarly, Park et al. [24] observed high rates of *Salmonella Typhimurium* and *S. Enteritidis* in febrile African adults with malaria.

Differences in bacterial isolates have also been observed. For example, Chau et al. [8] reported varied pathogens including *K. pneumoniae* and *H. influenzae*, while Ukaga et al. [15] found a higher prevalence of Gram-negative organisms like Pseudomonas and Klebsiella. These discrepancies may result from local microbial ecology or different pathophysiological pathways, such as variations in immune response and intestinal permeability [16].

### Antibiotic susceptibility patterns

*Salmonella typhi* showed high sensitivity to ciprofloxacin and levofloxacin. *Staphylococcus aureus* showed moderate susceptibility to doxycycline, azithromycin, and clindamycin but was resistant to gentamicin and minocycline. *Streptococcus* spp. was highly susceptible to clindamycin and cefepime but resistant to tetracycline.

These results differ from Akinyemi et al. [25], who reported reduced fluoroquinolone susceptibility among Salmonella in Lagos. Popoola et al. [26] also found high multidrug resistance in Salmonella and Staphylococcus isolates among febrile Nigerian patients, 68.5% of whom were children. Egbe and Enabulele [27] highlighted the efficacy of ceftriaxone and ceftazidime against Klebsiella spp., which was not commonly isolated in our setting. Such variations in susceptibility patterns are likely due to geographic differences and evolving antibiotic stewardship practices.

### Strengths and limitations

This is the first study, to our knowledge, to assess concomitant bacteremia among adults with severe malaria in Uganda. However, its single-center design and short study period may limit the generalizability of the results.

## Conclusion and recommendations

Bacteremia was present in over one in seven patients with malaria. Key associated factors included CNS symptoms, low peripheral oxygen saturation, hyperparasitaemia, and leucocytosis. *Salmonella typhi, Staphylococcus aureus*, and *Streptococcus* spp. were the most common isolates. *Salmonella typhi* was highly sensitive to ciprofloxacin and levofloxacin; *S. aureus* showed moderate sensitivity to doxycycline, azithromycin, and clindamycin but was resistant to gentamicin.

Patients with severe malaria should be assessed for bacteremia risk. Those with the above clinical features should be prioritized for empirical antibiotic treatment. In settings without culture access, ciprofloxacin combined with a penicillin may offer adequate empirical coverage for the most likely bacterial pathogens.

## Data Availability

The datasets generated and analyzed during the study are available from the corresponding author upon reasonable request.

## References

[1] Venkatesan P. News: WHO world malaria report 2024. Lancet Microbe. 2025;6(4):101073. 10.1016/j.lanmic.2025.101073 39923782

[2] Namuganga JF, et al. Malaria epidemiology in Uganda: the past and future. Malar J. 2021;20:176.33827592

[3] Means AR, et al. Malaria surveillance in Uganda: a spatial analysis. Malar J. 2014;13:432.25404126

[4] Were T, et al. Bacteremia in children with severe malaria in Kenya. Pediatr Infect Dis J. 2011;30:376–9.

[5] Ingrids M, et al. Bacteraemia in malaria among children in Tanzania. East Afr Med J. 2014;91:192–6.

[6] Nielsen MV, et al. Association of bacteremia with severe malaria. Am J Trop Med Hyg. 2015;93:15–21.

[7] United Nations Development Programme. Uganda human development report 2022. Kampala. UNDP. 2022.

[8] Chau TTH, et al. Bacteremia in Vietnamese adults with severe malaria. Clin Infect Dis. 2020;70:456–63.

[9] World Health Organization. Body mass index (BMI). WHO Global Health Observatory. 2025.

[10] Whelton PK, et al. 2017 guideline for the prevention, detection, evaluation, and management of high blood pressure in adults. J Am Coll Cardiol. 2017;71:e127-248. 29146535 10.1016/j.jacc.2017.11.006

[11] Wilairatana P, et al. Prevalence of bacteremia in malaria: a meta-analysis. J Trop Med. 2022;2022:1–12.

[12] Ricci JA. Malnutrition and poverty in sub-Saharan Africa. J Public Health Afr. 2012;3:e5.28299079

[13] Hanson J, et al. Bacterial co-infection in severe malaria. Trans R Soc Trop Med Hyg. 2021;115:30–6.32838408

[14] Nyein PP, et al. Bacteremia in malaria patients in Myanmar. Malar J. 2016;15:347.27387549

[15] Ukaga CN, et al. Concomitant bacteremia in Nigerian malaria patients. Niger J Parasitol. 2006;27:1–6.

[16] Donnelly CA, et al. Pathophysiological mechanisms linking malaria to bacteremia. Nat Microbiol. 2021;6:1120–8.

[17] White NJ. Neurological manifestations of malaria. Brain. 2022;145:368–80.

[18] White NJ. Severe falciparum malaria pathophysiology. Malar J. 2018;17:432.30454044

[19] Adebola A, et al. Airway and respiratory complications in cerebral malaria. Trop Med Int Health. 2014;19:225–31.

[20] Takem EN, et al. Hyperparasitemia and risk of bacterial infection. Malar J. 2014;13:49.24502679

[21] Babatunde SM, Adenuga AA. Neutrophils in malaria pathogenesis. Front Immunol. 2022;13:786–94. 10.3389/fimmu.2022.922377 PMC9367684 35967409

[22] Piyaphanee W, et al. Salmonella bacteremia in vivax malaria. Am J Trop Med Hyg. 2007;76:1044–6.

[23] Bhattacharya S, et al. Bloodstream infections in febrile adults in Kolkata. Indian J Med Microbiol. 2013;31:133–7.

[24] Park SE, et al. Salmonella bloodstream infections in Africa. Clin Infect Dis. 2016;62(Suppl 1):S4-10. 26933016 10.1093/cid/civ893 PMC4772835

[25] Akinyemi KO, et al. Antibiotic resistance in Salmonella. J Health Popul Nutr. 2007;25:194–8.

[26] Popoola M, et al. Antimicrobial resistance patterns in febrile Nigerian patients. Afr Health Sci. 2019;19:2315–23.

[27] Egbe CA, Enabulele OI. Fever of unknown origin and bacteremia in Nigeria. Afr J Clin Exp Microbiol. 2014;15:121–7.

